# Arsenic Accumulation of Realgar Altered by Disruption of Gut Microbiota in Mice

**DOI:** 10.1155/2020/8380473

**Published:** 2020-08-18

**Authors:** Wenfeng Xu, Shanshan Zhang, Wenqing Jiang, Shuo Xu, Pengfei Jin

**Affiliations:** ^1^Department of Pharmacy, Beijing Hospital, National Center of Gerontology, Institute of Geriatric Medicine, Chinese Academy of Medical Sciences, Beijing 100730, China; ^2^Assessment of Clinical Drugs Risk and Individual Application Key Laboratory, Beijing 100730, China

## Abstract

**Objective:**

To investigate the influence of gut microbiota on arsenic accumulation of realgar in mice.

**Methods:**

Mice were treated with antibiotics to form a mouse model of gut microbial disruption. Antibiotic-treated and normally raised mice were given 15 mg/kg, 150 mg/kg, and 750 mg/kg realgar by gavage and 0.2 mg/kg and 1 mg/kg arsenic solution by subcutaneous injection for 7 days. The concentration of arsenic in mice whole blood was determined by inductively coupled plasma mass spectrometry (ICP-MS). Arsenic accumulation in antibiotic-treated mice and normally raised mice was compared.

**Results:**

After exposure to low dose (15 mg/kg) and middle dose (150 mg/kg) of realgar, significantly, more arsenic was accumulated in the whole blood of antibiotic-treated mice compared to normally raised counterparts, which indicated that the disruption of gut microbiota could lead to higher arsenic load of realgar in mice. The homeostasis of gut microbiota was supposed to be disrupted by high dose (750 mg/kg) of realgar because after exposure to high dose of realgar, there was no significant difference in arsenic accumulation between antibiotic-treated and normally raised mice. Furthermore, arsenic solution was administered by subcutaneous injection to mice to investigate the influence of gut microbial differences on arsenic accumulation in addition to the absorption process, and there was no significant difference in arsenic accumulation between mice with these two different statuses of gut microbiota.

**Conclusions:**

Gut microbiota disruption could increase arsenic accumulation of realgar in mice.

## 1. Introduction

Realgar, which has been listed in Chinese Pharmacopoeia (ChP) since 1963, is a widely used mineral traditional Chinese medicine (TCM). It has significant therapeutic effects on various external and internal diseases, such as boils, lumps, furuncles, carbuncles, insect and snake bites, parasitoses, and convulsive epilepsy. In the recent years, realgar has been proved to have obvious antitumor activities in acute and chronic malignancies [1–4]. The main composition of realgar is arsenic disulfide (As_2_S_2_). Arsenic (As) is a ubiquitous metalloid, exposure to which may lead to serious health impacts including increased cancer risks and other adverse health effects [5–8]. Despite the therapeutic benefits of realgar, the potential toxic effects of arsenic have caused substantial concerns about the safety of realgar [9], as there have been some poisoning cases associated with excess doses or prolonged intake of realgar [10–13]. However, the arsenic toxicity and health risk of realgar to humans would be overestimated only by considering the total amount of arsenic because of the poor solubility of realgar in aqueous solutions [14, 15]. The fraction of As that is soluble in the gastrointestinal environment and available for absorption into the circulatory system is the key to study the potential toxicity of realgar [16, 17].

Gut microbiota is a complex community of microorganisms that dwell in the mammalian gastrointestinal tract [18, 19]. This community contains about 100 trillion microbes that belong to approximate 1000 species and encodes 100-fold more unique genes than the human genome [20, 21]. The gut microbiota is considered as important as the host's “second brain,” which is deeply involved in numerous host physiological processes, such as food digestion, immune system development, and xenobiotic biotransformation [22, 23]. In the recent years, the relationship between gut microbiota and human diseases has become a new research frontier [24]. Increasing evidences have suggested that gut microbiota is associated with a variety of human diseases, including obesity, diabetes, cardiovascular diseases, and so on [25–29]. In addition, external factors such as environment, bacterial infection, and antibiotics can affect the composition and diversity of gut microbiota [30].

For the past few years, there have been accumulating evidences of complex interactions between arsenic exposure and gut microbiota. On the one hand, arsenic exposure can perturb the normal gut microbiota composition, as well as its metabolic profiles [31–33]. On the other hand, gut microbiota can significantly impact arsenic biotransformation in mammals [34, 35] and, more importantly, the toxic effects of arsenic. The metabolic disorders in mice induced by arsenic exposure could be exacerbated by the perturbation of gut microbiota [36], and modulation of the gut microbiota might be an intervention strategy to reduce arsenic toxicity [37]. Furthermore, *in vivo* and *in vitro* experiments have demonstrated that gut microbiota can bioaccumulate arsenic and, thus, reduce the arsenic exposure of the host [34, 38].

Our previous *in vitro* studies have shown that gut microbiota could accumulate arsenic and, therefore, decrease the bioaccessibility of arsenic in realgar [39]. However, *in vitro* incubators cannot entirely simulate the complex environment of the gastrointestinal tract of mammals and cannot always reflect the true composition and metabolic activities of the gut microbiota [40]. At present, the effect of gut microbiota on arsenic exposure of realgar *in vivo* remains unclear. In this research, a mouse model with gut microbiota being disrupted from antibiotic treatment was employed to study the impact of gut microbiota perturbation on arsenic accumulation of realgar in mice. The concentration of arsenic in the whole blood of mice was determined by inductively coupled plasma mass spectrometry (ICP-MS), and the arsenic accumulation in gut microbiota-disrupted mice was compared with that in conventionally fed mice.

## 2. Materials and Methods

### 2.1. Chemicals and Reagents

Realgar was provided by Xi'an Yuelai Pharmaceutical Technology Co., Ltd. (Xi'an, China). Prior to the experiment, realgar was tested and confirmed to meet the standards of ChP (2015). Cefoperazone sodium was supplied by Shanghai yuanye Bio-Technology Co., Ltd. (Shanghai, China). The reference standard solution for arsenic with a concentration of 1000 mg/L was obtained from the National Institute of Metrology (Beijing, China). Nitric acid classified as the trace metal grade was purchased from Fisher Chemical (Fair Lawn, USA). ICP-MS tuning solution of mixed elements (Ce, Co, Li, Mg, Tl, and Y) with a concentration of 1 *μ*g/L and internal standard mix (100 mg/L of Bi, Ge, In, Li, Lu, Rh, Sc, and Tb) were provided by Agilent Technologies (Santa Clara, USA).

### 2.2. Animal Experiments

The animal experiments were approved by the Ethics Committee for Animal Experimentation of Beijing Hospital. Every effort was made to minimize the number of animals used and their suffering. Five-week-old male Kunming mice were purchased from Beijing HFK Bioscience Co., Ltd. (Beijing, China). All animals were maintained under standard environmental conditions (22–28°C, humidity 40–70%, and 12 h/12 h light-dark cycle).

The mice were allowed to drink and eat freely for 7 days to acclimatize to the environment and were, then, randomly allocated into two groups (60 mice/group). The mice in group A were treated with cefoperazone sodium in drinking water at the concentration of 0.5 mg/mL for 7 days to generate gut microbiota-disrupted animals as previously reported [37, 38, 41]. During this time, the mice in group B were given drinking water without antibiotics. Water containers were changed once a day to supply fresh antibiotics.

Then, group A and group B were divided into six groups (10 mice per group), respectively. Group A-1, control group of antibiotic-treated mice; antibiotic-treated mice exposed to realgar (group A-2, 15 mg/kg; group A-3, 150 mg/kg; group A-4, 750 mg/kg); antibiotic-treated mice exposed to arsenic solution (group A-5, 0.2 mg/kg; group A-6, 1 mg/kg); group B-1, control group of normal mice; normal mice treated with realgar (group B-2, 15 mg/kg; group B-3, 150 mg/kg; group B-4, 750 mg/kg); normal mice treated with arsenic solution (group B-5, 0.2 mg/kg; group B-6, 1 mg/kg). Realgar was administered intragastrically to mice once a day for 7 days. The dosages of realgar were set as 1, 10, and 50 times of the human therapeutic dose based on the literature [42] and our preliminary experiments. Arsenic solution was administered to mice once a day for 7 days by subcutaneous injection with the standard solution of arsenic (1 mg/mL) dissolved in normal saline. Antibiotic treatment still continued throughout realgar and As exposure to maintain gut microbiota disruption condition. All of the mice were anesthetized with ether and decapitated on day 8. The whole blood samples were taken and stored directly at −80°C before arsenic determination.

### 2.3. Sample Preparation for Arsenic Measurement

0.5 mL of the whole blood sample was added into the microwave digestion tube containing 2 mL of trace metal grade nitric acid, and then, the tube was heated in the water bath at 80°C for 1 h. The mixture was cooled to room temperature and digested in a CEM MARS x-press microwave digester (CEM Corporation, Matthews, USA) following a three-stage microwave digestion process. Stage 1: the temperature was raised from room temperature to 120°C in 10 min and kept at this temperature for 10 min. Stage 2: the temperature was, then, increased from 120°C to 140°C in 5 min and maintained at 140°C for 20 min. Stage 3: within 10 min, the temperature was raised to 180°C at which it was held for 20 min. The digested solution that has been cooled to room temperature was transferred to a 20 mL volumetric flask, and the remaining volume was replenished with water.

Blank solutions without blood samples need to be prepared and analyzed simultaneously in the same batch of samples. Samples with arsenic content beyond the linear range were diluted to suitable concentrations for ICP-MS detection.

### 2.4. Instrumentation and Analytical Methods

Arsenic concentrations in whole blood of mice were measured on the Agilent 7900 ICP-MS (Agilent Technologies International Japan, Ltd., Tokyo, Japan). Data acquisition was carried out on MassHunter 4.5 Workstation Software. Equipment tuning was conducted every day to ensure that the responses of Li, Y, and Tl are at least 3000, 10000, and 6000 counts, respectively, and the values of CeO/Ce and Ce^2+^/Ce are at most 2% and 3%. The specific operational parameters are shown in Table 1. The sample tube with an internal diameter (ID) of 1.02 mm was used to inject samples and standard solutions to the ICP-MS nebulizer. The internal standard mix (Section 2.1) was diluted with 5% (v/v) HNO_3_ to obtain the internal standard solution (0.01 mg/L of Bi, Ge, In, Li, Lu, Rh, Sc, and Tb), which was introduced into the ICP-MS through the internal standard tube (0.19 mm, ID) during the entire measurement process.

### 2.5. Method Validation

The method for the determination of arsenic in whole blood of mice established in this experiment was validated in accordance with the Guideline on Bioanalytical Method Validation (Guideline 9012) of ChP [43].

#### 2.5.1. Linearity

The arsenic calibration curve was evaluated using the calibration standards with concentrations of 0.1, 1, 2.5, 5, 10, and 20 ng/mL, which were obtained by continuous dilution of the standard solution of As with 5% HNO_3_. The calibration curve was established by plotting the ratio of counts per second (cps) of As to Ge (internal standard) against the corresponding concentrations of As.

#### 2.5.2. Accuracy, Precision, and Recovery

The accuracy and precision of the intra- and interbatch were assessed by analyzing five replicate samples from the same batch and three different batches. The replicate samples with the target concentrations of 5, 10, 400, and 600 ng/mL were prepared by adding arsenic standards to the blank mice whole blood. Arsenic is an element originally existing in mice blood. The blank blood collected from mice was mixed together to maintain the consistency for subsequent use [44]. The recovery was verified by comparing the cps of As in the whole blood of mice with that in the standard solution of the same concentration. Quintuplicate samples were validated at four concentrations, respectively.

#### 2.5.3. Stability Test

The effects of storage conditions on the stability of As in the whole blood of mice were evaluated by analyzing three independent samples at two validation concentrations. The samples were stored at −80°C for 14 and 60 d for short-term and long-term stability researches. Post-preparative stability was evaluated by storing the samples at 4°C and room temperature (28 ± 2°C) for 24 h.

### 2.6. Statistical Analysis

All quantitative data are expressed as mean ± SD. The independent samples *t*-test in SPSS 17.0 software (SPSS Inc., Chicago, USA) was applied to perform the statistical analysis. The statistical significance was set at a value of *p* < 0.05.

## 3. Results and Discussion

### 3.1. Method Validation

The calibration curve was *Y* = 0.3213*X* + 0.0091. The correlation coefficient (*r*) was 0.9996, indicating a good linear relationship throughout the concentration range of 0.1–20 ng/mL for arsenic. The limit of quantitation (LOQ) automatically generated by MassHunter software was 0.026 ng/mL, which was sensitive enough for the study. The accuracy and precision data are summarized in Table 2. In summary, the intra- and interbatch accuracy and precision were investigated at four arsenic levels (5, 10, 400, and 600 ng/mL). The intrabatch precision RSD was between 3.28% and 8.54%, and the interbatch precision RSD ranged from 3.77% to 9.45%. The relative error (RE) values of the four QC levels were −7.80%, −5.30%, −0.59%, and −1.34%, which showed that good accuracy (acceptable ±20% at the LOQ level and ±15% at the other levels) was obtained. The results of the recovery test which was conducted at four different arsenic levels ranged from 93.25% to 98.28%. The results of the stability test are displayed in Table 3. Stability studies showed that arsenic was stable in mice whole blood under the preservation and preparation conditions.

### 3.2. Arsenic Determination in Mice Whole Blood

**Figure 1:**
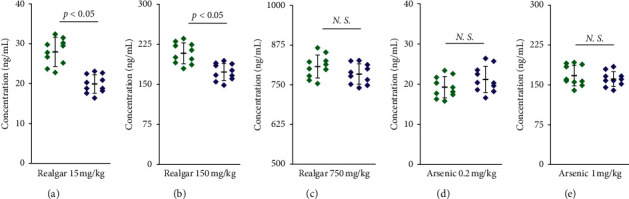
Arsenic concentrations in whole blood of antibiotic-treated mice (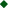) and normally raised mice (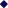) after administration of 15 mg/kg (a), 150 mg/kg (b), and 750 mg/kg (c) of realgar by gavage for 7 days and 0.2 mg/kg (d) and 1 mg/kg (e) of arsenic solution by subcutaneous injection for 7 days (*n* = 10, *N*.*S*.: no significant difference).

Since most of the absorbed arsenic accumulate in the form of stable complexes with thiol-containing proteins, such as hemoglobin in red blood cells, it is more scientific to use whole blood rather than serum or plasma to evaluate the arsenic accumulation of realgar [45, 46]. In this study, the whole blood arsenic content as the health risk assessment was compared between gut microbiota-disrupted mice and conventionally raised mice in order to investigate the influence of gut microbiota on the accumulation of arsenic in realgar. The results of arsenic accumulation in mice whole blood after administration of three doses of realgar by gavage and two doses of arsenic solution by subcutaneous injection are shown in Figure 1.

After multiple oral gavage of low dose (15 mg/kg) and middle dose (150 mg/kg) of realgar, significantly increased arsenic was accumulated in the whole blood of antibiotic-treated mice compared to normally raised counterparts, indicating that gut microbial disruption can significantly increase the arsenic exposure of realgar in mice (Figures 1(a) and 1(b)). This result is consistent with a previous study, in which groups of mice were exposed to 25 and 100 *μ*g/mL sodium arsenate (Na_2_HAsO_4_) in drinking water and more arsenic was found to retain in the liver and other organs of gut microbiota-disrupted mice than normally treated counterparts [38]. Gut bacteria was reported to be able to absorb arsenic through ion channels in the cell membrane [47], retaining more arsenic in the gastrointestinal tract and, thus, reducing the amount of arsenic available to the host. Consistently, significantly less arsenic was detected in the feces of mice with disrupted gut microbiota compared to conventionally fed mice exposed to an equivalent dose of arsenic [34, 38]. In our previous *in vitro* experiments, the gut microbiota was also found to bioaccumulate soluble arsenic from realgar and, thus, reduce the bioaccessibility of arsenic in realgar [39]. However, in order to further verify, *in vivo*, that the abovementioned differences in arsenic accumulation in mice mainly came from the influences of gut microbial differences during the absorption process, two levels of arsenic solution were subcutaneously injected into mice to eliminate the effects from the absorption process. The results (Figures 1(d) and 1(e)) indicated that there was no significant difference in arsenic accumulation between mice with these two different statuses of gut microbiota, which further confirmed the influences of gut microbiota on arsenic accumulation of realgar in mice mainly occurred in the absorption process.

Unlike the results of the low- and middle-dose realgar group, after exposure to high dose (750 mg/kg) of realgar, the levels of accumulated arsenic were not significantly different between antibiotic-treated mice and normally raised mice (Figure 1(c)). This result may be related to the changes in gut microbial community caused by high-dose realgar treatment. Some endogenous metabolites, such as lactate, pyruvate, and hippurate, which are cometabolites of the host and gut microbiota, were found to be altered by realgar exposure [48, 49]. Alterations in cometabolites of the host and gut microbiota were previously reported as the result of gut microbial perturbations [50, 51]. In terms of the composition at the phylum level, Firmicutes and Bacteroidetes were the two major phyla in the gut microbiota of mice, followed by Verrucomicrobia, Actinobacteria, Proteobacteria, and Tenericutes [33, 42]. Realgar treatment decreased the abundance of Firmicutes and increased the abundance of Bacteroidetes in a dose-dependent manner [42]. Several genera in Firmicutes, such as *Lactobacillus* and *Faecalibacterium*, were observed to decrease following arsenic exposure [42, 52]. *Lactobacillus* was beneficial in minimizing or preventing the adherence of xenobiotics to the surface of intestinal mucosa [53]. *Faecalibacterium prausnitzii* belonging to the genus *Faecalibacterium* is a useful species associated with microbial stability during arsenic exposure [38]. The genus *Bacteroides* in Bacteroidetes is related with the metabolism of bile acids, which are cholesterol derivatives synthesized in the liver [49]. Gut microbial perturbations caused by arsenic exposure were reported to affect the homeostasis of primary and secondary bile acid profiles as those induced by antibiotic treatment [33, 54]. The abundance of Proteobacteria and the genus *Enterobacter* in Proteobacteria increased with the increase of the dose of realgar [42]. The increased abundance of the phylum Proteobacteria was considered as a marker for an imbalanced or unstable gut microbial community and a potential diagnostic signature of dysbiosis and criterion for disease [55]. The homeostasis of gut microbiota could compensate for the influence of low-dose and medium-dose realgar exposure, but could not resist the microbial disorder caused by high dose of realgar. Finally, the strains in gut microbiota that can bioaccumulate arsenic need to be discovered and further studied. Gut microbiota has complex and profound effects on many physiological processes in the host. In this study, mice were administrated with antibiotics in an attempt to disrupt their gut microbiota. However, the complex effects of antibiotics on mice cannot be completely excluded and need to be further explored in the future.

## 4. Conclusions

The present study employed an antibiotics-treated mouse model to examine the influence of gut microbiota on arsenic accumulation of realgar. Arsenic concentration in mice whole blood as the potential health risk indicator was determined by ICP-MS. The results showed gut microbiota disruption could increase arsenic accumulation of realgar in mice. This work provided a selectable research perspective into the toxicity of realgar.

## Figures and Tables

**Table 1:** ICP-MS operating parameters.

Parameter	Value
RF power	1580 W
RF matching	1.2 V
Sampling depth	10.0 mm
Nebulizer gas	1.04 L/min
Makeup gas	—
Nebulizer pump	0.1 rps
S/C temperature	2°C
Acquisition mode	Spectrum
Peak pattern	3 points
Replicates	3
Sweeps per replicate	100
Monitored elements for calculations	^75^As and ^72^Ge
Integration time per mass	1.0 s for ^75^As, 0.3 s for ^72^Ge
Uptake speed	0.3 rps
Uptake time	40 s
Stabilization time	40 s

**Table 2:** Precision, accuracy, and recovery of total arsenic in mice whole blood.

Theoretical concentration (ng/mL)	Found (ng/mL) (mean ± SD, *n* = 5)			Overall mean (ng/mL)	Intrabatch RSD (%) (*n* = 15)	Interbatch RSD (%) (*n* = 15)	RE (%) (*n* = 15)	Recovery (%) (mean ± SD, *n* = 5)
	Batch 1	Batch 2	Batch 3					
5	4.45 ± 0.38	4.64 ± 0.49	4.74 ± 0.47	4.61	8.54	9.45	−7.80	93.25 ± 4.53
10	9.61 ± 0.54	9.42 ± 0.96	9.38 ± 0.73	9.47	5.58	7.53	−5.30	96.90 ± 2.41
400	393.76 ± 25.84	398.00 ± 15.52	400.40 ± 7.19	397.65	6.56	4.23	−0.59	97.27 ± 1.47
600	582.80 ± 19.14	600.32 ± 26.30	592.80 ± 22.25	591.97	3.28	3.77	−1.34	98.28 ± 1.12

**Table 3:** Stability of arsenic analysis in mice whole blood (mean ± SD, *n* = 3).

Theoretical concentration (ng/mL)	Recovery (%)			
	At −80°C for 14 days	At −80°C for 60 days	Post‐preparative at 4°C for 24 h	Post‐preparative at room temperature for 24 h
10	92.93 ± 3.23	90.67 ± 4.39	96.27 ± 4.67	98.27 ± 3.45
800	98.92 ± 1.87	95.30 ± 1.57	98.03 ± 5.62	97.63 ± 4.12

## Data Availability

The data used to support the findings of this study are available from the corresponding author upon request.
